# Frugale Zahnmedizin – Ressourceneinsparungen mit Fokussierung auf Kernfunktionen und Patientenbedürfnisse

**DOI:** 10.1007/s00103-021-03377-y

**Published:** 2021-07-09

**Authors:** Hans Jörg Staehle

**Affiliations:** Poliklinik für Zahnerhaltungskunde der Klinik für Mund‑, Zahn- und Kieferkrankheiten, Universitätsklinikum Heidelberg, Im Neuenheimer Feld 400, 69120 Heidelberg, Deutschland

**Keywords:** Frugale Zahnmedizin, Frugale Interventionen, Frugale Innovationen, Pseudofrugalität, Zahnlückenschluss mit direkten Kompositkunststoffrestaurationen, Frugal dentistry, Frugal interventions, Frugal innovations, Pseudofrugality, Tooth gap closure with direct composite resin restorations

## Abstract

Die frugale Zahnmedizin leitet sich vom Begriff „frugal“ (sinngemäß: einfach, sparsam, nutzbar, tauglich) ab. Sie richtet ihr Augenmerk auf zahnärztliche Interventionen, die im Vergleich zu Behandlungsalternativen zum einen mit einer Kostenreduktion verbunden sind und sich zum anderen auf zahnmedizinische Kernfunktionen unter Berücksichtigung der Patientenbedürfnisse konzentrieren. Die Weltgesundheitsorganisation (WHO) hat 2020 auf die Dringlichkeit hingewiesen, kosteneffiziente und erschwingliche Interventionen zu priorisieren.

Vor diesem Hintergrund erfolgen in diesem Beitrag eine Einordnung und eine Bestandsaufnahme zur frugalen Zahnmedizin. Es wird der Frage nachgegangen, inwieweit sich ihre Ziele substanziieren lassen. Anhand von Literaturquellen und einer exemplarischen klinischen Einzeldarstellung werden Optionen der frugalen Zahnmedizin aufgezeigt. Zudem werden für etliche zahnmedizinische Fächer Einzelbeispiele potenzieller frugaler Interventionen und Innovationen genannt und erläutert. Gravierende Defizite gibt es in der systematischen Entwicklung und Implementierung der frugalen Zahnmedizin. Es sind daher noch zahlreiche Anstrengungen in Forschung, Lehre und Patientenversorgung erforderlich.

**Zusatzmaterial online:**

Zusätzliche Informationen sind in der Online-Version dieses Artikels (10.1007/s00103-021-03377-y) enthalten.

## Abstract (en)

Frugal dentistry is derived from the term “frugal” (meaning simple, economical, usable, suitable). It focuses on dental interventions, which on one hand leads to a cost reduction compared to treatment alternatives and on the other hand focuses on dental core functionalities taking into account the patient’s needs. In 2020, the World Health Organization (WHO) highlighted the urgency to prioritize cost-effective and affordable interventions.

Against this background, a classification and review of frugal dentistry is carried out in this article. To what extent its objectives can be substantiated is investigated. Based on literature sources and an exemplary clinical presentation, options for frugal dentistry are shown. In addition, examples of potential frugal interventions and innovations are named and explained for a number of subject fields in dentistry. There are serious deficits in the systematic development and implementation of frugal dentistry. Numerous efforts in research, teaching, and patient care are therefore still required.

## Einleitung

Mundgesundheit hat ihren Preis! Bei manchen Maßnahmen scheint er eher günstig, bei anderen eher hoch. Epidemiologische und gesundheitsökonomische Betrachtungen dazu wurden u. a. von Niederman et al. vorgestellt [1]. Die Weltgesundheitsorganisation (WHO) hat 2020 auf die Dringlichkeit hingewiesen, kosteneffiziente und erschwingliche Innovationen/Interventionen zu priorisieren [2]. Vor diesem Hintergrund erfolgt in diesem Beitrag eine Einordnung und Bestandsaufnahme zur frugalen Zahnmedizin [3–5]. Zunächst wird erläutert, welchen Einfluss zahnmedizinische Innovationen unter Betrachtung des Kostenaspekts auf die Mundgesundheit der Bevölkerung haben können. Der Begriff der Frugalität und verschiedene diesbezügliche Anforderungen in der Zahnmedizin werden dargestellt. Eine exemplarische klinische Einzeldarstellung und Beispiele für potenzielle frugale Interventionen in verschiedenen zahnmedizinischen Fächern sollen die Optionen der frugalen Zahnmedizin veranschaulichen. Abschließend wird die Rolle von Kompositkunststoffen in der frugalen Zahnmedizin diskutiert.

## Zahnmedizinische Innovationen und ihr Einfluss auf die Mundgesundheit

Die Erhaltung oder Wiederherstellung der Mundgesundheit ist von einem Zusammenspiel endogener und exogener Einzelfaktoren abhängig. Auch wenn man in der Zahnmedizin lange das persönliche Arzt-Patienten-Verhältnis in den Vordergrund der Betrachtungen stellte, ist es heute unbestritten, dass in der Regel eine Kombination (semi)kollektiver und individueller Interventionen am besten zur Mundgesundheit beiträgt. Auf den Wandel von einer rein biomedizinischen Sichtweise hin zu einer stärkeren Fokussierung auf soziale Determinanten der Mundgesundheit und Public-Health-Strategien haben verschiedene Autoren, z. B. Peres et al. [6] und Sheiham et al. [7] aufmerksam gemacht.

Klassischerweise unterscheidet man zwischen Gesundheitsförderung, Krankheitsvorbeugung und Behebung bereits eingetretener initialer oder fortgeschrittener Schäden (einschließlich ihrer Diagnostik). Dabei stehen sich Leistungsempfänger und -erbringer gegenüber. Die „Leistungsempfänger“ sind teilweise durch heterogene Merkmale gekennzeichnet (u. a. in ihren Lebens‑, Bildungs- und Einkommensverhältnissen, Verhaltensgewohnheiten, Erwartungen, allgemeingesundheitlichen Gegebenheiten). Ähnlich ist es bei den „Leistungserbringern“, denen ebenfalls ein variables Portfolio (aus Qualifikation, Geräte‑, Instrumenten- und Materialienausrüstung, Honorarfluss usw.) mit mannigfaltigen Einflussnahmen auf Struktur‑, Prozess- und Ergebnisqualität zur Verfügung steht. Nicht nur „harte“ Daten, sondern auch „weiche“ Kriterien (Persönlichkeitsmerkmale, Vorlieben, Modeerscheinungen, „Zeitgeist“, gesellschaftlicher Status usw.) können bei Leistungsempfängern wie auch -erbringern viel dazu beitragen, ob und wie der jeweils aktuelle wissenschaftliche Kenntnisstand umgesetzt wird. Auch Gegebenheiten wie „asymmetrische Informationen“ zwischen Arzt und Patient und die daraus teilweise beeinflusste Nachfragesituation (Supplier‑/Provider-induced Demand) können hier genannt werden [8]. Sowohl innerhalb einer Population als auch zwischen verschiedenen Populationen existieren hinsichtlich der anzutreffenden (Mund‑)Gesundheitsstandards und -bedürfnisse Unterschiede, wie in der klassischen Arbeit von Bradshaw [9] über die diversen Arten von „Bedarf“ bereits vor 50 Jahren aufgezeigt wurde. Was in dem einen Milieu als „normal“ angesehen wird, kann in dem anderen als zu viel („utopisch“) oder zu wenig („inakzeptabel“) gelten.

Gemeinhin wird unterstellt, dass die Mundgesundheit umso höher sei, je mehr Mittel in die Lehre, Forschung und Patientenversorgung fließen. Allerdings sind solche Automatismen nicht zwangsläufig zu erwarten. Neue Interventionen können, müssen aber nicht zu einer Verbesserung der Mundgesundheit führen. Meistens handelt es sich nicht um eine einzige Intervention, sondern um Interaktionen verschiedener Bausteine, die zu einem bestimmten Ergebnis führen. Dies macht es zuweilen schwierig, den Anteil einer einzigen Maßnahme am Gesamtgeschehen zu definieren.

Um hier in erster Näherung eine Bewertung von Innovationen vornehmen zu können, orientiert man sich meist an den allgemein anerkannten, gängigen Praktiken. Eine Innovation kann im Vergleich dazu die Mundgesundheit verbessern, verschlechtern oder überhaupt nicht beeinflussen, und zwar bei erhöhten, geringeren oder gleichbleibenden Kosten. Für alle in Tab. 1 genannten 9 Möglichkeiten gibt es Beispiele aus dem zahnmedizinischen Spektrum. Allerdings sind solche Vergleiche in der Praxis zuweilen wenig nützlich, da sich die Frage, ob etwas mehr oder weniger Nutzen bringt, nicht immer mit einer einfachen Ja-Nein-Aussage beantworten lässt.

**Table 1:** 

	a) Erhöhte Kosten	b) Gesenkte Kosten	c) Kostenneutralität
1) Kein Einfluss auf Mundgesundheit	1a) Kostensteigernde Interventionsänderungen ohne Einfluss auf Wirkung und/oder Akzeptanz	1b)* Kostensenkende Interventionsänderungen ohne Einfluss auf Wirkung und/oder Akzeptanz*	1c) Kostenneutrale Interventionsänderungen ohne Einfluss auf Wirkung und/oder Akzeptanz
2) Bessere Mundgesundheit	2a)* Kostensteigernde Interventionsänderungen mit positivem Einfluss auf Wirkung und/oder Akzeptanz*	2b)* Kostensenkende Interventionsänderungen mit positivem Einfluss auf Wirkung und/oder Akzeptanz*	2c) *Kostenneutrale Interventionsänderungen mit positivem Einfluss auf Wirkung und/oder Akzeptanz*
3) Schlechtere Mundgesundheit	3a) Kostensteigernde Interventionsänderungen mit negativem Einfluss auf Wirkung und/oder Akzeptanz	3b) Kostensenkende Interventionsänderungen mit negativem Einfluss auf Wirkung und/oder Akzeptanz	3c) Kostenneutrale Interventionsänderungen mit negativem Einfluss auf Wirkung und/oder Akzeptanz

Kursiv hervorgehoben sind Auswirkungen, die für die frugale Zahnmedizin relevant sind

So kann z. B. ein neues Mundpflegeprodukt oder Medikament eine bessere Kontrolle mikrobieller Plaque bewirken, aber u. U. gleichzeitig nicht nur die Zahnhartsubstanz vermehrt angreifen, sondern sogar das ganze „Biotop“ der Mundhöhle verändern. Hier wäre im Sinne einer Nutzen-Risiko-Abwägung zu klären, welche Vorteile unter Hinnahme der Nachteile in ihrer Summation zur Erhöhung der Mundgesundheit beitragen. Mit einer technischen „Sensation“ zur Restaurierung von Zähnen lassen sich möglicherweise gewisse Verbesserungen erzielen, aber der Aufwand ist u. U. dermaßen hoch, dass er in keinem günstigen Verhältnis zum Zusatznutzen steht.

Andererseits gibt es Innovationen, die in ihrer Anfangsphase einen Zusatzaufwand mit sich bringen, nach einer mit mehr oder weniger starken Rückschlägen verbundenen Erprobungsphase dann doch noch zu einem klinisch relevanten positiven Nettoeffekt führen. Ein bekanntes Beispiel dafür sind intraoral eingebrachte Restaurationen auf Kompositkunststoffbasis, die sich erst nach einer von zahlreichen Irrwegen belasteten Entwicklungs- und Erprobungsphase von mehreren Jahrzenten durchsetzen konnten (Näheres siehe unten).

Bei solchen Überlegungen steht zwar der Aspekt einer gesundheitlichen Bewertung im Vordergrund, aber es geht fast immer auch um die Kosten, von denen der Zugang zu einer zahnmedizinischen Versorgung abhängig sein kann. Nicht selten sind neue Technologien im Vergleich zu etablierten Verfahren teurer und damit nicht unbedingt für jedermann erschwinglich. In manchen Ländern/Settings werden sie unter Umständen erst gar nicht angeboten (z. B. mangels ausreichend geschulten zahnmedizinischen Personals oder geeigneter Ausrüstungen). Hier kommen die Optionen einer „frugalen Zahnmedizin“ ins Spiel.

## Begriffsbestimmung Frugalität

Der Begriff „frugal“ lässt sich auf das lateinische Wort „frugalis“ zurückführen. Es bedeutet ursprünglich „von (Feld)früchten stammend“ (im Sinn von „karg“, „genügsam“; [10]). Andere Charakterisierungen sind „einfach“, „sparsam“, „anspruchslos“, „bescheiden“, „nutzbar“ und „tauglich“ [11–13].

In der Betriebswirtschaftslehre findet sich von Herstatt und Tiwari folgende Definition: „Frugale Innovationen können als neue Produkte und Dienstleistungen verstanden werden, die den Einsatz von materiellen und finanziellen Ressourcen im kompletten Produktlebenszyklus von der Entwicklung und Produktion bis hin zur Nutzung und Entsorgung zu minimieren suchen und die Besitz- bzw. Nutzungskosten bei gleichzeitiger Gewährleistung akzeptabler Sicherheits- und Qualitätsstandards beim Verwender substantiell reduzieren“ [12]. Für frugale Innovationen werden folgende Eckpunkte benannt:

- Nicht zu verwechseln mit bloßen Billigprodukten/-dienstleistungen (keine reine Low-Cost-Strategie), aber auch keine Fokussierung auf Premiumsegmente
- Es gibt eine Nachfrage nach Produkten/Dienstleistungen mit folgenden Merkmalen:
  - „nachhaltig“ („sustainable“)
  - „bezahlbar“ („affordable“)
  - „adäquat“/„gut genug“ („good enough“)
- Zielgruppe: Menschen, die Produkte/Dienstleistungen ohne übertriebene Funktionalität („ohne Schnickschnack“) nachfragen, die haltbar und ressourcenschonend sind
- Es geht nicht nur um Menschen, die teure Produkte/Dienstleistungen nicht zahlen *können,* sondern vor allem auch um solche, die zu teure, unnötige und evtl. sogar mit Nachteilen verbundene Produkte/Dienstleistungen nicht erwerben (zahlen) *wollen *[12]

Weyrauch nennt 3 Hauptkriterien für frugale Innovationen:

- Substanzielle Kostenreduktion
- Konzentration auf Kernfunktionalitäten
- Optimiertes Leistungsniveau

Es handelt sich laut Weyrauch dagegen *nicht* um frugale Innovationen, wenn die Kostenreduktion nur sehr gering ist, Modifikationen von Produkten oder Dienstleistungen ohne fundierte Analyse von Bedürfnissen der anzusprechenden Menschen vorgenommen werden bzw. diesen nicht optimal Rechnung getragen wird [13].

Vor dem Hintergrund dieser Beschreibung sind frugale Produkte/Dienstleistungen nicht nur für ärmere, sondern auch für reichere Länder von Bedeutung. Eine pauschale Definition von „frugal“ ist insofern schwierig, als bestimmte Methoden (z. B. die sog. atraumatische Füllungsbehandlung) für ärmere Länder eine Verbesserung, für reichere hingegen eine Verschlechterung ergeben könnten. Wichtig erscheint es, mit differenzierten Angeboten größere Zielgruppen, die sich dafür interessieren bzw. die davon profitieren könnten, anzusprechen [11].

## Zahnmedizinische Anforderungen an Frugalität und Beispiele für frugale Strategien

In der Zahnmedizin bedürfen vor allem solche Entwicklungen einer näheren Betrachtung, die bei gesenkten oder neutralen Kosten einen gleichen oder besseren Gesamtnutzen oder bei erhöhten Kosten einen besonders relevanten und ausgeprägten Gesamtnutzen erzielen (Tab. 1).

Unter übergeordneter Berücksichtigung ethischer Aspekte (Patientenautonomie/Erfüllung von Patientenbedürfnissen, Benefizienz, Non-Malefizienz, Gerechtigkeit; [14]) ist hinsichtlich zahnmedizinischer Anforderungen (zunächst unabhängig von den Kosten) unter anderem folgendes Profil zu nennen:

1. Die Interventionen sollen möglichst belastungsarm und schonend (z. B. substanzschonend) sein.
2. Sie sollen möglichst keine negativen lokalen und systemischen Einflüsse entfalten.
3. Sie sollen auch bei Patienten mit Grunderkrankungen anwendbar sein.
4. Sie sollen grundlegende Qualitätsmerkmale erfüllen, wobei die Kriterien je nach zahnärztlicher Fachdisziplin sehr unterschiedlich ausfallen können (eine Schmerzbehandlung folgt anderen Bedürfnissen als ein Eingriff zur Verbesserung des Aussehens). Aktuelle Standards (z. B. hinsichtlich Funktionalität, Aussehen und Haltbarkeit restaurativer Interventionen) sind in der Literatur jeweils im Detail definiert.
5. Sie sollen hinreichend praktikabel sein (erlernbar auf der Grundlage einer klar umschriebenen Vorgehensweise).

Zu diesen ersten 5 Punkten kommen für die Frugalität noch weitere Aspekte hinzu:

6. Sie sollen möglichst wenig Ressourcen verbrauchen (Vermeidung von aufwendigen Techniken, kostspieligen Geräteanschaffungen, Anhäufungen von zu entsorgenden, potenziell umweltbelastenden Materialien usw.).
7. Sie sollen möglichst in einem überschaubaren zeitlichen Rahmen stattfinden (ohne Aspekte von Monitoring und Nachsorge zu vernachlässigen).
8. Sie sollen eine Option für Patienten bieten, die die ihnen genannten Kosten für eine Behandlungsmaßnahme nicht aufbringen können oder wollen und die sich (aus welchen Gründen auch immer) für weitere als die ihnen angebotenen Behandlungsalternativen interessieren. Es geht hier um „bezahlbare“ Interventionen (nicht „billig“, aber auch nicht stark kostentreibend, „sozial verträglich“), die manchen Menschen als „gut genug“ erscheinen (individuelle Erwartungen und Bedürfnisse erfüllend, gute Nutzen-Risiko-Relation unter Berücksichtigung der Verhältnismäßigkeit des Mitteleinsatzes).

Frugalität spielt seit jeher eine Rolle, weshalb hier nicht nur frugale *Innovationen*, sondern auch bewährte frugale *Interventionen* aufgeführt und unter dem Begriff „frugale Zahnmedizin“ zusammengefasst werden. Kern et al. sprechen in diesem Zusammenhang von frugalen Methoden [3]. Wie aus Tab. 2 hervorgeht, können für alle zahnmedizinischen Fachgebiete Beispiele zur Diskussion gestellt werden. Die Tabelle ist nicht vollständig, sondern ließe sich noch in hohem Umfang erweitern. Dies würde allerdings den Rahmen dieses Beitrags sprengen, weshalb auch auf die Nennung von Einzelreferenzen verzichtet wird.

**Table 2:** 

	Fachgebiet	Strategien/Eingriffe
*1*	*Fachübergreifend*	Ausbau von breitenwirksamer mundgesundheitlicher Aufklärung entsprechend dem aktuellen wissenschaftlichen Kenntnisstand, wenn relevante Wissenslücken identifiziert wurden Nutzung technologischer Weiterentwicklungen (z. B. digitale Techniken/Telemedizin/künstliche Intelligenz) in (Früh‑)Diagnostik und Risikoprofilerstellung als Grundlage von fachübergreifenden Entscheidungsunterstützungen für präventive und therapeutische Interventionen, wenn Vorteile (z. B. hinsichtlich Funktionalität, Übersichtlichkeit, Dokumentation und Zeitbedarf) gegenüber bisher üblichen Vorgehensweisen überwiegen Monitoring (einschließlich systematisch geplanter Routineuntersuchungen bzw. Recalls) anstatt Ad-hoc-Interventionen, wenn die angetroffenen Situationen dies zulassen
*2*	*Zahnerhaltungskunde*	
2.1	Präventive Zahnheilkunde (hier: Zahnhartsubstanzen)	Einbeziehung kollektivpräventiver Interventionen (z. B. Fluoridierung von Speisesalz) anstatt ausschließlich individuellpräventiver Interventionen Bei individuellpräventiven Interventionen Differenzierung zwischen karies- und nichtkariesbedingten Zahnhartsubstanzschäden mit alters- und bedarfsgerechten Empfehlungen (z. B. zu Fluoridangebot, Ernährung, Mundhygiene, Versiegelungen, Schutzvorrichtungen) statt Einsatz pauschal festgelegter Maßnahmen
2.2	Restaurative Zahnheilkunde	Direkte Restaurationen statt indirekter Restaurationen bei Zahnhartsubstanzerkrankungen unterschiedlicher Ursachen, wenn Nutzen-Risiko- und Nutzen-Kosten-Abwägungen dies rechtfertigen Reparieren von defekten Restaurationen aller Art, statt diese neu anzufertigen bzw. zu ersetzen Direkter Lückenschluss statt kieferorthopädischer, prothetischer oder chirurgischer/implantologischer Eingriffe, wenn Nutzen-Risiko- und Nutzen-Kosten-Abwägungen dies rechtfertigen
2.3	Kinderzahnheilkunde	Direkte Restaurationen statt vorgefertigter oder indirekter Restaurationen, z. B. bei Molaren-Inzisiven-Hypomineralisation (MIH), wenn Nutzen-Risiko- und Nutzen-Kosten-Abwägungen dies rechtfertigen
2.4	Endodontologie und dentale Traumatologie	Pulpotomie als Alternative zur Pulpektomie, wenn Nutzen-Risiko- und Nutzen-Kosten-Abwägungen dies rechtfertigen Zapfen- statt Stiftverankerung bei der postendodontischen restaurativen Versorgung, wenn die Voraussetzungen für stabile Fixierungen vorliegen Intentionelle Replantation (mit extraoraler Wurzelspitzenresektion, retrograder Wurzelkanalfüllung und Reinsertion (Wiedereinfügung) des betreffenden Zahnes in die originäre Alveole) als Alternative zur Extraktion mit anschließender prothetischer bzw. restaurativer Lückenversorgung oder einem kieferorthopädischen Lückenschluss, wenn Nutzen-Risiko- und Nutzen-Kosten-Abwägungen dies rechtfertigen Autotransplantation eines Zahnes als Alternative zu einer implantatgestützten Restauration, einer Brückenversorgung oder einem kieferorthopädischen Lückenschluss, wenn Nutzen-Risiko- und Nutzen-Kosten-Abwägungen dies rechtfertigen
2.5	Parodontologie	Ausbau von aktiven Mundhygienetrainingsmaßnahmen zugunsten rein passiver Interventionen (Hilfe zur Selbsthilfe, einschließlich individualisierten, digital gesteuerten und ggf. visualisierten Selbstmonitorings) Systematisches Monitoring (einschl. Diagnostik) und Risikoprofilerstellung als Grundlage für gezieltere nichtchirurgische und chirurgische parodontale Interventionen
*3*	*Zahnärztliche Prothetik*	
3.1	Kronen- und Brückenprothetik	Fakultative Verfolgung des Prinzips der verkürzten Zahnreihe statt obligater Zahnersatzausdehnung bis einschließlich der zweiten Molaren Reparaturen von festsitzendem Zahnersatz statt Neuanfertigung Bestimmte Formen von (u. U. CAD-CAM-gefertigten) Adhäsivbrücken statt konventioneller Brücken, wenn Nutzen-Risiko- und Nutzen-Kosten-Abwägungen dies rechtfertigen
3.2	Herausnehmbare Prothetik	Nutzung von Werkstoffen (einschl. monolithischer Materialien) mit verbesserten Eigenschaften bei gleichzeitig einfacherer Verarbeitung, wenn Nutzen-Risiko- und Nutzen-Kosten-Abwägungen dies rechtfertigen
3.3	Funktionslehre	Nichtinvasive Maßnahmen wie Physiotherapie und Selbstmonitoring bei Funktionsstörungen (einschließlich Habits bzw. myofunktionellen Störungen) anstelle invasiver Eingriffe, wenn Nutzen-Risiko- und Nutzen-Kosten-Abwägungen dies rechtfertigen
*4*	*Oralchirurgie*	
4.1	Allgemeine zahnärztlich-chirurgische Eingriffe	Neuerungen in der Indikationsstellung und technischen Durchführung von operativen Eingriffen (z. B. Weisheitszahnentfernungen, endochirurgische Interventionen)
4.2	Implantologie	Modifikationen (Anzahl, Größe, Form, Standardisierung usw.) von Implantaten bzw. Verbindungsteilen (Abutments) zur Verbesserung bei gleichzeitiger Vereinfachung der Vorgehensweise (z. B. kurze Implantate statt Augmentationen, interne statt externe Sinusliftinterventionen, Reduktion der Implantatanzahl bei zahnlosem Unterkiefer mit genügend Knochenangebot), wenn Nutzen-Risiko- und Nutzen-Kosten-Abwägungen dies rechtfertigen (Anmerkung: Implantatversorgungen werden häufig als besonders kosten-, zeit- und materialintensive Therapieoption angesehen, allerdings gibt es auch hier frugale Methoden, z. B. die bessere und zugleich einfachere Fixierung einer Unterkiefertotalprothese mit einem Einzelimplantat)
*5*	*Kieferorthopädie*	
5.1	Allgemeine Kieferorthopädie	Neuerungen in der Indikationsstellung und technischen Durchführung der kieferorthopädischen Behandlung beim Erwachsenen – selektive kieferorthopädische Behandlungsmaßnahmen Erweitertes Spektrum an Behandlungsapparaturen ermöglicht in der individuellen Situation ausgewogene Nutzen-Risiko-Abwägung Hoch individuelle Mechaniken zur Mobilisation verlagerter Zähne und zur Umsetzung selektiver 3‑D-Zahnbewegungen
8.2	Interdisziplinäre Kieferorthopädie	Lokale eingesetzte Extrusionsapparaturen zum Zahnerhalt ansonsten schwer zu versorgender Zähne als präprothetische Maßnahme oder nach Frontzahntrauma

Bei manchen Innovationen kann, wie oben ausgeführt, nicht auf Anhieb entschieden werden, ob es sich um echte frugale Entwicklungen im Sinne einer Konzentration auf Kernfunktionalitäten mit optimiertem Leistungsniveau bei substanzieller Kostenreduktion oder um „pseudofrugale“ Entwicklungen handelt, bei denen dies nicht der Fall ist. Entsprechende Nachweisführungen sind nicht nur in der (Zahn‑)Medizin, sondern auch bei industriellen Verbrauchsprodukten verschiedenster Sparten mitunter schwierig [13]. Hinzu kommt der Umstand, dass es sich bei einer frugalen Innovation nicht immer um eine technische Neuentwicklung handeln muss. Die Innovation kann auch darin bestehen, dass die Patientenbedürfnisse besser identifiziert und bereits bekannte Techniken an diese adaptiert werden.

### Beispiel: frugale Intervention zum Lückenschluss

**Figure 1:**
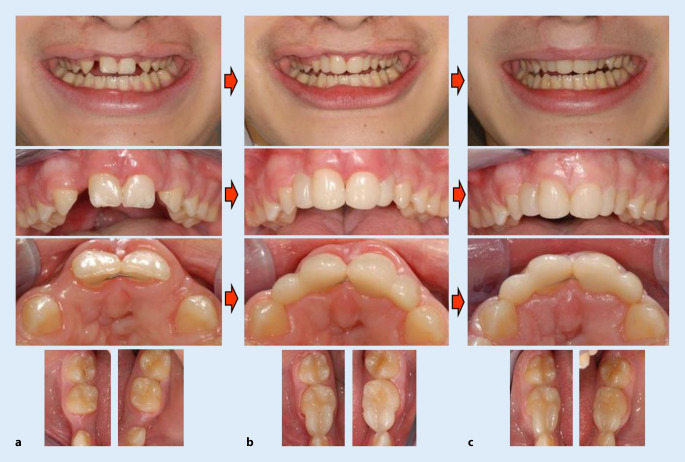

An einem Einzelbeispiel der Lückenversorgung eines 16-jährigen Patienten mit einer vorbehandelten Lippen-Kiefer-Gaumenspalte werden Überlegungen zur Vorgehensweise unter Berücksichtigung frugaler Aspekte vorgestellt (Abb. 1 und Onlineabbildungen 1 und 2). Zur Zeit der Erstvorstellung beim Verfasser (Poliklinik für Zahnerhaltungskunde des Universitätsklinikums Heidelberg) bestanden 2 Einzelzahnlücken im Oberkiefer (seitliche Schneidezähne) und 2 Einzelzahnlücken im Unterkiefer (Prämolaren). Unter der Berücksichtigung der komplexen Vorbehandlungen, der teilweise ungünstigen Knochenverhältnisse, der lädierten Zahnhartsubstanzen der beiden mittleren Schneidezähne, der kurzen oberen Eckzahnkronen, des geringen Patientenalters und weiterer Faktoren schien eine Lückenversorgung mit Implantaten oder konventionellen Brücken nicht unproblematisch. Deshalb wurde der Lückenschluss mit einer neuen minimal-invasiven Restaurationsmethode (direkte metall-, keramik- und glasfaserfreie Zahnanhänger aus Kompositkunststoff) hergestellt. Dadurch konnten mit einem überschaubaren Aufwand die Gebissverhältnisse hinsichtlich des Aussehens, der Hygienefähigkeit (Reinigungsmöglichkeit mittels Interdentalraumbürsten) und der Funktion verbessert werden.

Im Anschluss daran wurde mit Interventionen der Mund‑, Kiefer- und Gesichtschirurgie (Klinik und Poliklinik der MKG-Chirurgie des Universitätsklinikums Heidelberg) eine Oberlippenplastik vorgenommen. Näheres zum technischen Vorgehen findet sich bei Staehle [15]. Die restaurative Versorgung funktioniert seit 4,5 Jahren problemlos. Da es sich um eine Neuentwicklung ohne ausreichende Langzeiterfahrungen handelt, kann keine abgesicherte Prognose zur Haltbarkeit gegeben werden. Aufgrund des minimal-invasiven Vorgehens könnte im Bedarfsfall jederzeit die Ausgangssituation wiederhergestellt werden und einer alternativen Behandlungsoption Platz machen. (Ein weiteres Beispiel für eine frugale Intervention zum Lückenschluss zeigt Onlineabbildung 3.)

### Werden die zahnmedizinischen Anforderungen erfüllt?

Die einzelnen Behandlungsschritte wurden in den letzten Jahren mehr und mehr differenziert, vor allem was die Versorgung im Seitenzahnbereich angeht [16–21]. Im Folgenden wird aufgezeigt, inwieweit bei den genannten Beispielen die oben genannten 8 zahnmedizinischen Anforderungen für frugale Methoden erfüllt sind:

#### Anforderung 1: Möglichst nicht- oder minimal-invasiv (Schonung oraler Strukturen).

Was den hier beschriebenen Lückenschluss betrifft, so kann der Anforderung 1 heute in hohem Umfang Rechnung getragen werden, allerdings zu dem Preis, dass die Haftflächen auf weite Teile der Pfeilerzähne ausgedehnt werden müssen und Überdimensionierungen zur Erzielung hinreichender Schichtdicken zuweilen nicht zu vermeiden sind. Dies kann zu Einschränkungen im Aussehen, im Tragekomfort und in der Hygiene führen, wobei die ersten Erfahrungen jedoch zeigen, dass eine für den Patienten ansprechende Erscheinungsform und Oberflächengestaltung (u. a. zum Schutz des Parodontalgewebes einschließlich Reinigungsfähigkeit) realisierbar sind. Weitere Hinweise zu Vor- und Nachteilen finden sich bei Frese und Staehle [22].

#### Anforderung 2: Keine negativen lokalen und systemischen Einflüsse.

Bei den hier verwendeten Präparaten sind Nebenwirkungen (z. B. Allergien) nicht auszuschließen. Nach aktuellen Kenntnissen sind diese aber selten. Bezüglich der lokalen Reaktionen ist bei Überdimensionierungen fast immer mit einem erhöhten Risiko für Plaqueretention zu rechnen. Dieses Problem lässt sich durch die Verwendung spezieller Hilfsmittel (Interdentalraumbürsten) angehen. Darüber hinaus schaffen die Restaurationen bei korrekter Gestaltung ein Widerlager für die Interdentalraumbürsten, sodass die Hygienefähigkeit sogar verbessert werden kann. Präzisierte Vorschläge zur Auswahl von Interdentalraumbürsten stehen inzwischen zur Verfügung [23, 24].

#### Anforderung 3: Medizinisch anwendbar auch bei Patienten mit Grunderkrankungen.

Bei bestimmten allgemeinmedizinischen Grunderkrankungen (z. B. Diabetes mellitus, immunologischen Erkrankungen, Krankheiten, die Medikamente wie Bisphosphonate oder Chemotherapie erforderlich machen) gibt es einige absolute oder relative Kontraindikationen für invasive Versorgungen wie Implantationen. In solchen Fällen kann unter Umständen auf die hier genannten Versorgungsformen ausgewichen werden. Diese Aspekte sind u. a. für die Gerontostomatologie von Relevanz. Die Versorgung älterer und pflegebedürftiger Personen stellt weltweit eine große Herausforderung dar. Es gibt zwar auch Senioren, die besonders aufwendige und teure „Premiumversorgungen“ nachfragen. Da jedoch die Anzahl multimorbider und dementsprechend eingeschränkter Menschen steigt, gewinnt die Option für einfache und möglichst belastungsarme Lösungen an Bedeutung.

#### Anforderung 4: Erfüllung grundlegender Qualitätsmerkmale.

**Figure 2:**
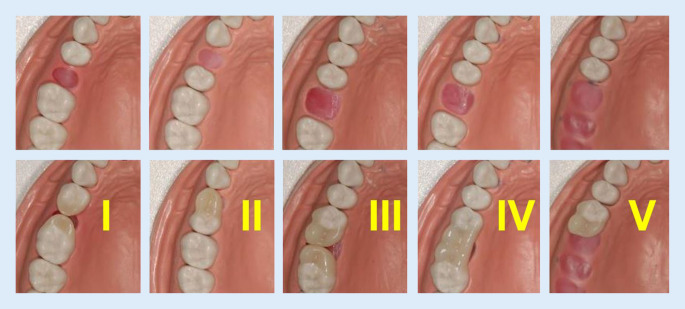

Zu Qualitätsmerkmalen gehören unter anderem Funktionalität, Aussehen und Haltbarkeit. Bei den hier zu bewertenden Innovationen steht der Nachweis, dass sie diese Anforderungen erfüllen, noch weitgehend aus. Es liegen nur wenige Fallbeobachtungen und Untersuchungen vor. Eine Studie von 2015 bezieht sich auf den Lückenschluss mittels direkter Zahnverbreiterungen [25]. Eine weitere Studie, bei der auch weitere Kategorien direkter Kompositkunststoffrestaurationen mit einbezogen wurden (Abb. 2), befindet sich in Vorbereitung [26]. Die Ergebnisse lassen zwar auf stabile und haltbare Versorgungen schließen, die den Erwartungen Rechnung tragen. Allerdings ist die Datenlage im Vergleich zu der Vielzahl von Studien über andere Versorgungsoptionen (z. B. Brücken oder Implantate) gering.

#### Anforderung 5: Hinreichend praktikabel.

Neue Methoden, die sich von gängigen Praktiken unterscheiden, können anfangs zu Umstellungen und umfangreichen Einarbeitungen zwingen. Die hier beschriebenen neuen Versorgungsformen sind zwar auf der Basis einer klar beschriebenen Vorgehensweise prinzipiell erlernbar, aufgrund der momentan noch sehr anspruchsvollen Handhabung bedarf es aber einer Einarbeitungs- und Übungsphase. Für die Zukunft sind Erleichterungen durch Nutzung einfacher digitaler Techniken zu erwarten.

#### Anforderung 6: Ressourcenschonung.

Der Anforderung 6, nämlich möglichst wenig Ressourcen zu verbrauchen (Vermeidung von aufwendigen Techniken, kostspieligen Geräteanschaffungen, zu entsorgenden Materialien usw.), lässt sich mit den neuen Restaurationsverfahren gut Rechnung tragen. Die Prozeduren sind zwar schwierig zu erlernen (siehe oben), aber nach einer Übungsphase hinreichend gut umzusetzen (siehe auch Anforderung 8). Man benötigt keine speziellen Geräteinvestitionen, die über die übliche Grundausrüstung für Kompositkunststoffanwendungen hinausgehen, und es fallen auch nur wenige zu entsorgende Materialien an.

#### Anforderung 7: Überschaubarer zeitlicher Rahmen.

Die für den direkten Lückenschluss erforderlichen Maßnahmen können in einem überschaubaren zeitlichen Rahmen (in wenigen Sitzungen) stattfinden. Auch die Anforderungen von Monitoring und Nachsorge sind ohne großen Aufwand zu erfüllen.

#### Anforderung 8: Begrenzte Kosten.

Die hier beschriebenen Interventionen sind nicht nur aus (zahn-)medizinischen Gründen (geringe Invasivität im Vergleich zu konventionellen prothetischen Eingriffen, keine Herausforderungen für den Gesamtorganismus, wie z. B. die Immunabwehr bei implantologischen Eingriffen usw.) vielversprechend. Darüber hinaus haben sie das Potenzial für eine Kostenreduktion. Dies gilt aber momentan nur für geübte und erfahrene Behandler. Bei fehlender Routine ist das Verfahren nicht nur mit dem Risiko von Qualitätsmängeln behaftet, sondern erfordert einen immensen Zeitaufwand, der über etablierte Verfahren sogar hinausgehen kann. Es sind noch etliche Anstrengungen erforderlich, um das Verfahren einfacher zu gestalten.

## Die Rolle von Kompositkunststoffen in der frugalen Zahnmedizin

Anhand der wechselvollen Geschichte dentaler Kunststoffe und Adhäsive lässt sich exemplarisch aufzeigen, welche Schwierigkeiten bei der Entwicklung und Verbreitung frugaler Interventionen auftreten können.

Kunststoffe wurden 1930 in die Zahnmedizin eingeführt [27]. Sie leisteten einen Beitrag dazu, größere Bevölkerungsteile zu erschwinglichen Kosten mit Prothesen zu versorgen. Schwieriger war die etwa 10 Jahre später (um 1940) eingeleitete Entwicklung von Kunststoffen für konservierende Zwecke. Auch hier bildeten neben zahnmedizinischen Anforderungen (z. B. zahnfarbenes Aussehen und Biokompatibilität) Aspekte der Frugalität (hier: Ressourcenschonung und Kostenersparnis) eine Triebfeder. Man wollte direkte, im Mund herstellbare Kunststoffrestaurationen anbieten, da damit eine Breitenversorgung der Bevölkerung eher realisierbar erschien als mit den teureren laborgefertigten Werkstücken (Inlays, Kronen usw.). Grundlegende Entdeckungen zur Möglichkeit, Kunststoffe an Zahnhartsubstanzen minimal invasiv anzukleben, folgten wiederum etwa 10 Jahre später (um 1950). Der Weg zu direkt eingebrachten, stabilen und adhäsiv verankerten Kunststoffrestaurationen (später als Kompositkunststoffrestaurationen bezeichnet) war von etlichen Misserfolgen begleitet. Erst ab den 1970er- und 1980er-Jahren standen einigermaßen brauchbare Materialien und Techniken (vor allem für den Frontzahnbereich) zur Verfügung. Der Seitenzahnbereich blieb noch längere Zeit hart „umkämpft“, denn hier erwiesen sich Kompositkunststoffe als wesentlich problembehafteter als die seinerzeit noch am meisten verbreiteten Amalgame.

Der Durchbruch der Kompositkunststoffe gelang nicht nur durch materialtechnische Verbesserungen, sondern wurde auch durch Änderungen in der Nachfrage („Bedürfnisse“) beeinflusst. Dentale Metalle (allen voran Amalgame) wurden besonders intensiv zum Ende des 20. Jahrhunderts durch öffentlichkeitswirksame Angstkampagnen in Misskredit gebracht, wobei nicht immer ganz klar war, wer die Aktionen aus welcher Interessenkonstellation heraus entfachte bzw. förderte [28, 29]. Wissenschaftliche Argumentationen, die vor Überschätzungen toxischer Wirkungen mit daraus resultierenden Nocebovorstellungen (Nocebo, lat.: „ich werde schaden“) warnten, konnten jedenfalls die negative Stimmungslage nicht entscheidend ändern. Die als Amalgamalternativen oder -ersatz angebotenen Kompositkunststoffe waren zunächst allerdings keineswegs „frugal“, da sie sich wegen ihrer noch deutlich begrenzten Materialgüte und wegen der für den ungeübten Behandler schwierigeren Verarbeitbarkeit in einer ungünstigen Kombination („schlechter und teurer“, entsprechend der Einstufung 3a in Tab. 1) präsentierten. Dennoch sank in der Bevölkerung zunehmend die Akzeptanz für die bisher üblichen Versorgungsformen.

Diese Methode zur Steuerung von Nachfrageimpulsen hatte, so wissenschaftlich fragwürdig sie auch war, entscheidenden Anteil an der Verbreitung der Kompositkunststoffe. Die Hintergründe wurden in 2 Gutachten für den Sachverständigenrat für die Konzertierte Aktion im Gesundheitswesen der Jahre 1996 und 2002 beleuchtet [28, 29]. Die Schulungen der Zahnärzteschaft zur besseren Verarbeitung kompositkunststoffbasierter Restaurationsmaterialien wurden notgedrungen intensiviert und durch Honorarsteigerungen begleitet. Entrichtet wurden die Mehrkosten von den Leistungsempfängern, die infolge der entfachten Vergiftungsängste vor Metallen meist gerne dazu bereit waren.

Wirklich „frugal“ wurden direkte Kompositkunststoffrestaurationen erst später, als man bemerkte, dass sie erfolgreich in ein Terrain eindrangen, das zuvor durch die wesentlich teureren indirekten Versorgungsformen (Inlays, Kronen usw.) abgedeckt worden war. Als man dann auch noch daran ging, weitere Indikationsgebiete zu erschließen (z. B. Reparaturrestaurationen, postendodontische Aufbauten, Farb- und Formkorrekturen), wurden sie weltweit nahezu unverzichtbar. Obwohl die Zahnärzteschaft inzwischen über mehr Erfahrungen verfügt, kann die Entwicklung nicht als abgeschlossen betrachtet werden. Ein kritischer Blick in die Versorgungsrealität macht noch Schwächen von derartigen Restaurationen (Frakturen, Abplatzungen, Verschleißerscheinungen, Randmängel, Randkaries und vieles andere) deutlich. Gleichwohl kann davon ausgegangen werden, dass sich die Anwendungsgebiete weiter vergrößern werden.

Für die hier beschriebenen direkten Methoden zum Schließen von Zahnlücken sieht die Situation noch ernüchternd aus. Die Entscheidungsgrundlagen, ob und ggf. wie ein Lückenschluss erfolgen soll, sind variabel und zuweilen nicht klar begründet [30]. Auch seitens der Industrie sind kaum Aktivitäten zu verzeichnen, werkstoffkundliche Schwächen von Kompositkunststoffen (z. B. mangelnde Frakturfestigkeit), die eine Indikationsausweitung erschweren, zu beheben. Momentan gelten Implantate für den Lückenschluss vielfach als Mittel der ersten Wahl, aber auch andere Neuerungen (z. B hightechgefertigte Adhäsivbrücken) sind auf dem Vormarsch.

Es finden sich deshalb noch kaum Zahnärzte, die bereit sind, eine schwierig erlernbare Lowtechmethode bei gleichzeitig (zumindest in der Anfangsphase) geringerem Verdienst anzubieten. Die Situation könnte sich allerdings ändern, wenn kostspieligere Methoden wie Brücken oder Implantate für einen versorgungsrelevanten Anteil der Bevölkerung nicht mehr bezahlbar sind (z. B. bei einer länger anhaltenden wirtschaftlichen Rezession) und keine entscheidenden Erfolge bei der Behandlung von Implantatnebenwirkungen (z. B. Periimplantitiden = entzündungsbedingter Abbau des Knochenlagers um Implantate) erzielt werden. Kompositkunststoffe waren schon immer gut für „frugale“ Überraschungen. Vor diesem Hintergrund ist es nicht ausgeschlossen, dass der Lückenschluss mit direkt eingebrachtem Kompositkunststoff größere Verbreitung finden wird. Dies dürfte auch für momentan noch im Hintergrund stehende frugale Methoden anderer Fachdisziplinen gelten.

## Fazit und Ausblick

Um das Interesse an frugaler Zahnmedizin zu fördern, sind diverse Anstrengungen in Forschung, Lehre und Patientenversorgung erforderlich.

Es gibt momentan (abgesehen von Ausnahmen wie dem Innovationsfonds des Gemeinsamen Bundesausschusses (G-BA) mit Blick auf Verbesserungen der Kostenwirksamkeit) kaum Projekte, die dezidiert frugale Innovationen verfolgen. Allerdings können in nahezu jeder Disziplin Bereiche identifiziert werden, für die frugale Potenziale bestehen. Allein ihre Benennung und die Aufzählung der zur Umsetzung notwendigen Schritte seitens der beteiligten Akteure (Industrie, Forschungseinrichtungen usw.) kann zu einem Umdenken führen. Auch die Forschungsförderung durch vermehrte Ausschreibung öffentlicher Drittmittel für konkrete Projekte wäre hier zu nennen.

Der aktuelle Kenntnisstand zu frugalen Interventionen/Innovationen wird in der Lehre noch nicht systematisch vermittelt. Es wäre sinnvoll, Themen der frugalen Zahnmedizin in Lehrpläne bzw. Angebote prägradualer Ausbildungen sowie postgradualer Fortbildungen und Weiterqualifikationen aufzunehmen. In Verlautbarungen (wie Stellungnahmen, Empfehlungen, Richtlinien, Leitlinien usw.) öffentlicher und privater Einrichtungen (einschließlich Körperschaften, Verbände, Vereine, Fachgesellschaften usw.) findet sich bislang kaum etwas zu dieser Thematik. Hier wäre somit zunächst ein Bewusstsein für solche Richtungen zu schaffen.

In die Patientenversorgung fließen Überlegungen zu frugalen Interventionen seit jeher ein, allerdings weniger aus (zahn-)medizinischen Erwägungen heraus, sondern oft erst dann, wenn die Umsetzung von Behandlungsvorschlägen scheitert, z. B. an der Kostenfrage. Dies wird dann eher als „schicksalhaft“ angesehen und weniger als Motivation für eine Veränderung. Dabei gibt es durchaus eine mehr oder weniger latente Nachfrage von Patientenseite nach frugalen Interventionen, die allerdings mangels entsprechender Angebote der Zahnärzteschaft noch nicht befriedigt werden kann. Wenn auch seitens der Leistungserbringer und der Kostenträger die Thematik vermehrt bearbeitet wird, dürfte der Ruf nach frugalen Interventionen höhere Chancen als bisher haben, Gehör zu finden.

## Supplementary Information


