# Supplementary material for: Frugal dentistry—saving resources with a focus on core functions and patient needs
Source: Bundesgesundheitsblatt Gesundheitsforschung Gesundheitsschutz. 2021 Jul 9;64(8):1001–10. [Article in German] doi: 10.1007/s00103-021-03377-y (PMC8316237; doi:10.1007/s00103-021-03377-y)

Bundesgesundheitsblatt 8/2021

Elektronisches Zusatzmaterial zum Beitrag:

Frugale Zahnmedizin – Ressourceneinsparungen mit Fokussierung auf Kernfunktionen und Patientenbedürfnisse

Hans Jörg Staehle[1]

[1] Poliklinik für Zahnerhaltungskunde der Klinik für Mund-, Zahn- und Kieferkrankheiten des Universitätsklinikums Heidelberg, Heidelberg, Deutschland

Korrespondenzadresse
Prof. Dr. Dr. Hans Jörg Staehle
Poliklinik für Zahnerhaltungskunde der Klinik für Mund-, Zahn- und Kieferkrankheiten
Universitätsklinikums Heidelberg
Im Neuenheimer Feld 400
69120 Heidelberg
Deutschland
hansjoerg.staehle@med.uni-heidelberg.de

Inhalte:

Onlineabbildung 1: Patient mit chirurgisch und kieferorthopädisch vorbehandelter doppelseitiger Lippen-Kiefer-Gaumen-Spalte [15].
Onlineabbildung 2: Patient bei einer Kontrolle nach 5 Jahren im Alter von 21 Jahren (Fortsetzung von Abb. 1 im Artikel).
Onlineabbildung 3: Lücke regio 25 einer 40-jährigen Patientin (nähere Angaben [16]).

Onlineabbildung 1: Patient mit chirurgisch und kieferorthopädisch vorbehandelter doppelseitiger Lippen-Kiefer-Gaumen-Spalte [15].

a) Modelldarstellung zur Oberkiefer-Situation im Alter von 12 Jahren (beachte unter anderem die Position der Oberschneidezähne)
b) Modellsituation des Oberkiefers im Alter von 16 Jahren nach weiteren kieferorthopädischen Interventionen zum Zeitpunkt der Erstvorstellung für die Planung restaurativer Maßnahmen (Implantate?, Brücken?, direkte Zahnumformungen und -anhänger?)
c) Röntgenzahnfilm im Bereich der Oberkiefer-Schneidezähne mit Darstellung der Lücken und Knochendefekte
d) Klinische Darstellung der Oberkiefer-Frontzahnsituation mit kurzen Eckzähnen, Lücken im Bereich der seitlichen Schneidezähne und Hartsubstanzschäden der mittleren Schneidezähne, Situation beim Lachen (beachte auch die Form der Oberlippe). Es fehlen insgesamt vier Zähne (im Oberkiefer die seitlichen Schneidezähne, im Unterkiefer zwei Prämolaren). Nach Konsultation verschiedener Fachdisziplinen wurde die Entscheidung zur restaurativen Therapie mit direkten Zahnanhängern getroffen
e) Als Pfeilerzähne wurden im Oberkiefer die mittleren Schneidezähne ausgewählt. Hier: Der Zahn 11 wurde unter absoluter Trockenlegung (Kofferdam) mit Komposit-Kunststoff umgeformt (verlängert) und mit einem Distalanhänger versehen
f) verlängerter Zahn 11 mit Distalanhänger nach Feinkorrektur und Politur
g) analoges Vorgehen an Zahn 21
h) Röntgenkontrolle. Die Lücken im Unterkiefer wurden mit direkt reingebrachten Zahnanhängern (Kategorie II, siehe. Abb. 2 innerhalb des Artikels) geschlossen

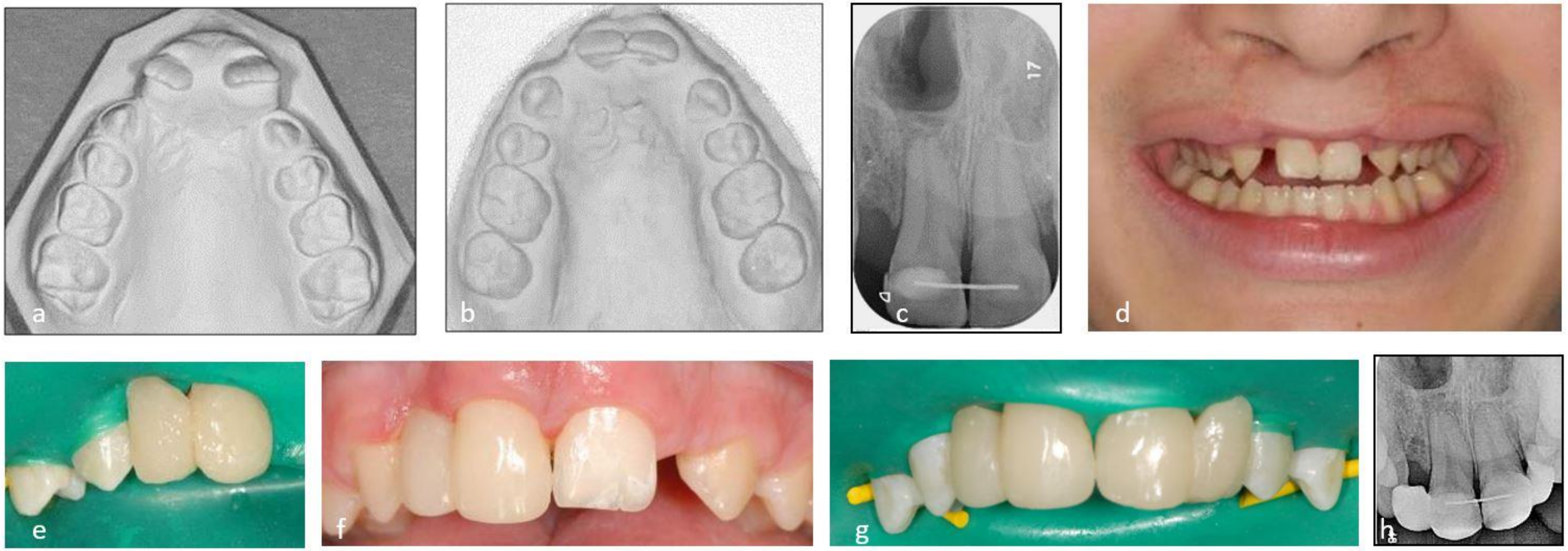

(Anmerkung: Ich bedanke mich bei Herrn Prof. Dr. Chr. Lux, Poliklinik für Kieferorthopädie der MZK-Klinik des Universitätsklinikums Heidelberg für die Erlaubnis, Abb. a wiedergeben zu dürfen. Alle anderen Abbildungen stammen vom Verfasser.)

Onlineabbildung 2: Patient bei einer Kontrolle nach 5 Jahren im Alter von 21 Jahren (Fortsetzung von Abb. 1 im Artikel).

d) und e) Situation im Oberkiefer
f) und g) Situation im Unterkiefer

Es finden sich keine Anhaltspunkte für relevante pathologische Veränderungen der Zahnhartsubstanzen, des Endodonts, des Parodonts einschließlich der Hygienefähigkeit (siehe e: Passage von Interdentalraumbürsten), des Aussehens oder der Kaufunktion

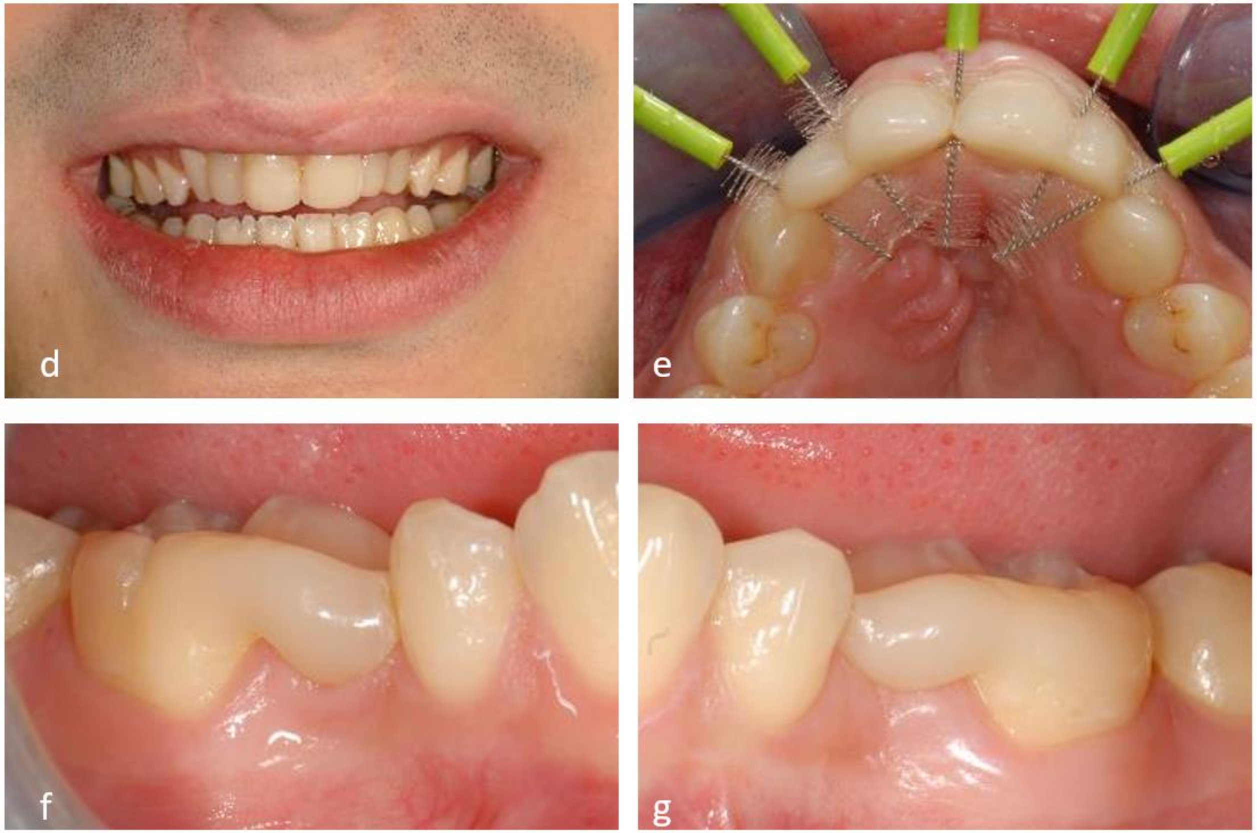

Onlineabbildung 3: Lücke regio 25 einer 40-jährigen Patientin (nähere Angaben [16]).

a) Ansicht von okklusal
b) Ansicht von lateral. Die Lücke ist beim Lachen sichtbar. Die Patientin fragt nach einer implantatfreien und möglichst gering-invasiven restaurativen Versorgung
c) die Hygienefähigkeit ist an den zur Lücke hingewandten Approximalflächen der Zähne 24 und 26 eingeschränkt. Es kommt zum Bluten nach Sondieren (hier: Situation an Zahn 26 mesial)
d) Röntgenbissflügel
e) Einfügen eines direkt hergestellten Zahnanhängers unter Kofferdam
f) nach okklusaler Justierung, Politur und Anpassung von Interdentalraumbürsten
g) fertiger Zahnanhänger von okklusal
h) fertiger Zahnanhänger von lateral
i) wegen verbesserter Hygienefähigkeit besteht jetzt kein Bluten mehr auf Sondieren
j) Röntgenkontrolle des Zahnanhängers
k) bis n) Kontrolle nach zwei Jahren: unauffällige Verhältnisse

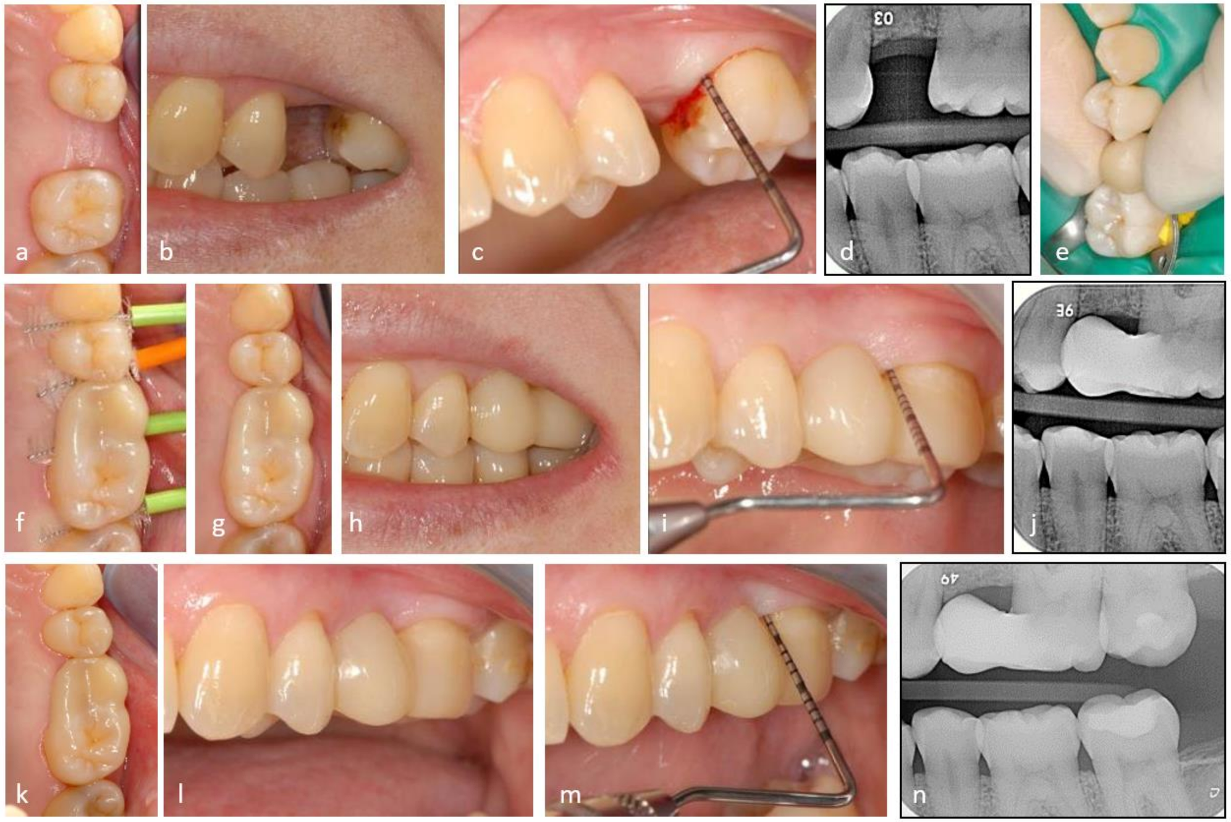

Supplement: Supplementary file 1 [file 103_2021_3377_MOESM1_ESM.pdf]
